# The legal dilemma of medical artificial intelligence in China: challenges to physicians' duty to inform and a typology-based response

**DOI:** 10.3389/fpubh.2025.1747635

**Published:** 2026-01-13

**Authors:** Chi Zhang

**Affiliations:** Law School, Tianjin University, Tianjin, China

**Keywords:** four-level classification system, liability allocation, medical artificial intelligence, physicians' duty to inform, right to informed consent

## Abstract

The rapid advancement of artificial intelligence is profoundly reshaping clinical practice and transforming the doctor–patient relationship from a traditional physician–patient dyad to a composite structure of “physician + AI–patient”. While this structural shift can improve diagnostic and therapeutic efficiency, it also poses unprecedented challenges to the normative design and legal application of the physician's duty to inform. In traditional settings, this duty has evolved from standardization to strictness and then to substantive requirements. With the introduction of medical AI, a fourth transformation toward a typology-based approach is urgently needed. Once AI participates in medical decision-making, this composite actor disrupts the trust relationship between physicians and patients, expands the scope of the physician's duty to inform, and blurs the allocation of legal responsibility. Moreover, existing AI classification schemes are misaligned with physicians' duty to inform. This article employs doctrinal analysis, case analysis, and comparative legal research. At present, international norms tend to construct legal and ethical frameworks around the core concept of “risk prevention”. In the field of medical AI, this article proposes that—based on the current state of development—a “cooperation” concept be added on the foundation of risk prevention. On the premise that medical AI should not be recognized as a legal subject, we construct a four-level classification system centered on “decisional autonomy” and the degree of impact on the physician–patient relationship, together with a corresponding tiered accountability regime. Through a tripartite linkage mechanism—decision-making authority, duty to inform, and liability allocation—we aim to achieve a dynamic balance between technological complexity and patients' informed consent, and to promote the orderly development of medical AI within the framework of the rule of law and ethics.

## 1 Introduction

China's development of medical AI started relatively late but has progressed very rapidly. Its applications have penetrated multiple stages of disease screening, diagnosis, treatment, and health management, demonstrating substantial potential to improve clinical efficiency, optimize resource allocation, and empower primary care. China's vast medical data resources and rich application scenarios have driven the rapid adoption and iteration of AI technologies in the medical field. In the Proposal of the CPC Central Committee on Formulating the Fifteenth Five-Year Plan for National Economic and Social Development (2026–2030), the nation clearly called for “accelerating innovation in digital and intelligent technologies such as artificial intelligence” and “fully implementing the ‘AI+' initiative to comprehensively empower all industries” (1). At the sectoral level, the National Health Commission and other authorities have issued a series of policy documents—including the Implementation Plan for the Digital and Intelligent Transformation of the Pharmaceutical Industry (2025–2030), the Action Plan for High-Quality Development of the Pharmaceutical Industry (2023–2025), the Action Plan for Manufacturing Digital Transformation, and the Opinions on Deepening Drug and Medical Device Regulatory Reform to Promote High-Quality Development of the Pharmaceutical Industry—all of which set clear requirements for medical AI. Medical AI can significantly improve surgical precision and flexibility, broaden physicians' surgical field of view, and make greater visualization and accessibility possible (2).

At the same time, the ambiguity introduced by AI involvement makes the fulfillment of the physician's duty to inform difficult. This duty reflects respect for patient autonomy and human dignity and is also a necessary condition for legitimizing medical interventions. A physician's duty to inform is the precondition for patients to exercise their right to informed consent—the process from the provider's disclosure to the patient's decision should be continuous, consisting of “disclosure” by medical staff and “decision-making” by the patient (3). However, with the introduction of AI-assisted diagnosis and treatment, the original physician–patient dyad has been disrupted and transformed into a composite dyad of “physician+AI–patient”. Changes in the number of actors and in the physician–patient relationship directly increase medical-legal risks and pose challenges to the physician's duty to inform. With AI involvement, physicians are very likely to expand the scope of disclosure, heighten projections of potential outcomes, and blur the allocation of responsibility as a form of self-protection. This not only heightens patient anxiety and deepens the information gap—making autonomous decision-making more difficult—but also reduces the duty to inform to a mere formality and could even generate ethical risks. Conversely, if physicians disclose information only within the bounds of their personal judgment, effectively distancing themselves from AI-assisted care, it will undermine physicians' professional ethics, intensify tensions in the doctor–patient relationship, and even increase risks to physicians' personal safety. Therefore, in the context of AI-assisted diagnosis and treatment, the duty to inform must be adjusted. Otherwise, serious legal and ethical issues are likely to arise.

## 2 Physicians' duty to inform in China's traditional clinical setting

Traditional medical ethics emphasizes information symmetry and the construction of trust between physicians and patients. Physicians must explain the treatment process, potential risks, alternative options, and other relevant information to patients to safeguard patients' ability to make autonomous decisions (4). Although medical professionals possess the expertise to make judgments and decisions, patients have the right to make autonomous decisions regarding their own rights to life, health, and bodily integrity, and medical professionals should grant full respect and protection to these rights.

### 2.1 Three transformations of physicians' duty to inform

China's legal doctrine on the duty to inform patients has undergone at least three major transformations. The first was standardization, wherein laws explicitly established physicians' duty to inform in order to safeguard patient autonomy. The second was strictness, which refined formerly coarse legal norms and introduced clear requirements for the scope of disclosure, disclosure procedures, and the responsible disclosing party. The third was substantiveness, shifting the previously formalistic disclosure standards to a more substantive approach, better suited to complex clinical practice. Now, with the advent of medical AI, a fourth transformation is on the horizon, with typology as its defining theme.

#### 2.1.1 The first transformation: from ambiguity to clarity

Article 33 of the Regulations on the Administration of Medical Institutions, promulgated in 1994, provides: “Where a medical institution performs surgery, special examinations, or special treatments, it must obtain the patient's consent, and shall also obtain the consent and signature of the patient's family members or related persons; where the patient's opinion cannot be obtained, it shall obtain the consent and signature of the patient's family members or related persons; where neither the patient's opinion can be obtained nor any family member or related person is present, or where other special circumstances arise, the attending physician shall propose a medical treatment plan and implement it after obtaining approval from the person in charge of the medical institution or an authorized responsible person”. This provision does not explicitly stipulate a duty to inform. Instead, it implicitly requires physicians to obtain the consent of the patient or the patient's family, thereby compelling physicians to fulfill their disclosure duty through the consent-and-signature mechanism. Because a family member's or related person's signature was required, the primary recipients of the physician's disclosure were, in practice, the patient's family or related persons rather than the patient.

Article 62 of the Implementing Rules of the Regulations on the Administration of Medical Institutions, promulgated in 1994, provides: “Medical institutions shall respect the patient's right to be informed of their own condition, diagnosis, and treatment. When performing surgery, special examinations, or special treatments, they shall provide the necessary explanation to the patient. If, due to the implementation of protective medical measures, it is not appropriate to explain the situation to the patient, the relevant situation shall be notified to the patient's family” (5). As these Implementing Rules have not been revised subsequently, they preserve the initial normative style of the physician's duty to inform. The rules use the term “respect” for the patient's right to informed consent, and use “explain” rather than “inform” with regard to the physician's duty, indicating a very low scope and degree of disclosure. When it is deemed unsuitable to inform the patient, only “notification” of the “family” is required, rather than obtaining the “explicit consent” of a “close relative” as mandated by the latest standards, revealing coarse definitions of “family” and a rudimentary disclosure procedure.

Article 26 of the Practicing Physicians Law of the PRC, promulgated in 1998, provides: “Physicians shall truthfully inform the patient or the patient's family of the patient's condition, but must take care to avoid causing adverse consequences to the patient. When a physician conducts experimental clinical medical treatment, it shall be approved by the hospital and the consent of the patient themself or their family shall be obtained”. This provision presents a broad requirement for practicing physicians to provide explanations, clarifying physicians' duty to inform and related precautions. However, the rule is rather vague overall and does not differentiate obligations based on specific circumstances.

In 2002, the State Council promulgated the Regulations on the Handling of Medical Accidents. Article 11 stipulates that, in the course of medical activities, medical institutions and their staff shall truthfully inform patients of their condition, medical measures, and medical risks, and shall promptly respond to patients' inquiries. However, they should avoid causing adverse consequences for the patient (6). Compared with earlier provisions, this article explicitly added “medical risks” to the disclosure scope, but it did not expressly refer to alternative treatment options. Its defining feature is the incorporation of a protective-therapeutic consideration—namely, that disclosure should be calibrated to avoid foreseeable harm to the patient.

#### 2.1.2 The second transformation: from roughness to refinement

Article 55 of the Tort Liability Law of the PRC, promulgated in 2009, stipulates: “Medical personnel shall, during diagnosis and treatment, explain the patient's condition and medical measures to the patient. Where surgery, special examinations, or special treatments are needed, medical personnel shall promptly explain medical risks, alternative treatment plans, and other such matters to the patient, and obtain the patient's written consent; if it is not appropriate to explain to the patient, the explanation shall be given to the patient's close relatives and their written consent obtained. If medical personnel fail to fulfill the above obligation and thereby cause harm to the patient, the medical institution shall bear liability for compensation” (7). This provision embodies the mid-stage style of the duty-to-inform, including “medical risks” and “alternative treatment plans” in the scope of disclosure and explicitly limiting the family members who can consent on the patient's behalf to “close relatives”. However, it uses the term “written consent” rather than “explicit consent”, not accounting for scenarios in which a patient in critical condition cannot provide written consent, or cases where close relatives refuse to sign due to fear of liability.

Article 13 of the Regulations on the Prevention and Handling of Medical Disputes, promulgated in 2018, stipulates: “Medical personnel shall explain the patient's condition and medical measures in the course of diagnosis and treatment. If surgery or clinical trials or other special examinations or treatments that carry certain dangers or may result in adverse consequences are needed, medical personnel shall promptly explain the medical risks, alternative treatment plans, and other relevant information to the patient, and obtain the patient's written consent. If the patient is unconscious or otherwise unable to make decisions autonomously, or if the patient's condition makes it inappropriate to provide an explanation, the situation shall be explained to the patient's close relatives and their written consent obtained. In urgent circumstances where neither the patient nor the patient's close relatives can provide an opinion, with approval from the person in charge of the medical institution or an authorized responsible person, the relevant medical measures may be implemented immediately” (8). This provision anticipates the special scenario of emergencies and adds an appropriate measure: in an emergency, medical measures can be immediately taken upon approval by the hospital's responsible person or designee. It demonstrates respect for human rights and a humanitarian concern for preserving life.

#### 2.1.3 The third transformation: from formality to substantiveness

Article 1219 of the Civil Code of the PRC (2020) explicitly provides for the physician's duty to inform. This duty can be analyzed on three levels: First, medical personnel must explain the patient's condition and medical measures during diagnosis and treatment. Second, if surgery, special examinations, or special treatments are required, medical personnel must promptly provide the patient with specific explanations of medical risks, alternative plans, and so forth, and obtain the patient's explicit consent. Third, if it is impossible or inappropriate to explain to the patient, the explanation must be given to the patient's close relatives and their explicit consent obtained (9). This article introduced three changes to Article 55 of the former Tort Liability Law: (a) it requires medical staff to provide patients with specific explanations of medical risks, raising the standard of disclosure; (b) it replaced “written consent” with “explicit consent”, shifting from a formal standard to a substantive one; and (c) in addition to scenarios where explanation is “not appropriate”, it added scenarios where explanation is “not possible”, making the rule more comprehensive. These changes signal the maturation of the physician's duty to inform.

Furthermore, Article 43 of the Mental Health Law of the PRC (2012, amended 2018) provides special provisions for patients with mental disorders, reflecting special care for these patients. Such patients may lack the capacity to consent, so in circumstances that could infringe upon their health rights, an additional procedural requirement is imposed: approval from the medical institution's ethics committee must be obtained (10). Article 32 of the Basic Healthcare and Health Promotion Law (2019) (11) and Articles 25 and 55 of the Physicians Law (2021) (12) reaffirm the physician's duty to inform as established in the Civil Code and delineate sanctions for violations. Article 32 of the Regulations on the Administration of Medical Institutions (revised 2022) (13) was updated to align with the latest Civil Code provisions, maintaining consistency with the Civil Code. At this stage, the legal norms governing the duty to inform are relatively mature. The revised provision merges Civil Code Articles 1219 and 1220 into a single composite clause covering “general circumstances + exceptional circumstances”.

### 2.2 The scope and exceptions of physicians' duty to inform

Medical malpractice liability in China is based on a fault liability system, and the presence of “medical fault” is one of the essential elements in establishing liability for medical harm. Currently, there are three scenarios in which medical negligence is determined in China: (a) whether the duty to inform and the patient's right to informed consent were breached; (b) whether the duty of diagnosis and treatment was breached; and (c) whether there is a presumption of fault under special circumstances. A violation of the duty to inform is one such scenario.

First, we must distinguish a physician's duty to inform from the duty to treat, as the two are evaluated from different perspectives. Typically, whether a physician has fulfilled the duty of treatment is judged from the physician's perspective using a “reasonable physician standard”—essentially, the level of care expected of a similarly situated physician under equivalent conditions. In contrast, whether a physician has fulfilled the duty to inform is judged from the patient's perspective, focusing on whether the patient was provided with information that is materially important to an informed consent decision (14). The criterion of whether the physician provided information of material importance is still somewhat abstract. Currently, the Australian Rogers standard offers a well-developed interpretation of “materiality” and “significance”. This standard evaluates two dimensions—an “objective limb” and a “subjective limb”. The objective limb represents a proactive duty to warn directed toward the average patient, while the subjective limb represents a reactive duty to warn directed toward a specific patient or special patient population (15). The Rogers standard considers both the physician's and the patient's perspectives, creating a balanced and flexible framework: it preserves the patient's opportunity to proactively inquire, yet provides physicians with a relatively clear method to fulfill their disclosure obligations.

With regard to the scope of the physician's duty to inform, a physician is not required to obtain patient consent in every situation. The Civil Code establishes two levels of disclosure obligations: a general duty to inform and a special duty to inform. The general duty to inform only requires the physician to “explain the patient's condition and medical measures” and does not necessitate obtaining explicit consent. Only when it is necessary to “perform surgery, special examinations, or special treatments”—three specific scenarios—does the physician need to obtain consent from the patient or their close relatives. This more stringent duty to inform is the special duty to inform, a distinction that better aligns with social realities and life's practical patterns. Article 88(3) of the Regulations on the Administration of Medical Institutions and its Implementing Rules further clarifies “special examinations” and “special treatments” as diagnostic or therapeutic activities that meet any of the following criteria: (a) examinations or treatments that carry certain risks and could produce adverse outcomes; (b) examinations or treatments that, due to the patient's unique constitution or a critical condition, might pose adverse outcomes or dangers; (c) experimental examinations or treatments; or (d) examinations or treatments that may impose a substantial financial burden on the patient. In disclosing information, the physician should adopt the perspective of a reasonable patient.

For instance, even if a medical professional considers it common knowledge that a tonsillectomy must be performed before a styloidectomy, or that surgery for early endometrial cancer invariably requires removal of the uterus and its appendages, a patient might not be aware of these facts. Failure to inform the patient of such facts would breach the duty to inform (16). In 1999, patient C developed left eyelid ptosis after undergoing surgical removal of a lipoma in the left eye at a hospital. The ptosis was identified as a surgical complication, and the hospital's diagnostic and treatment approach was found to be appropriate, meaning no medical malpractice was present. However, the hospital's pre-operative discussion record with the patient's family did not clearly disclose that the surgery could result in the specific complication of “levator muscle rupture”. The court found that the hospital, given its professional knowledge, should have been able to foresee this risk but failed to sufficiently inform the patient. Thus, the physician's performance of the duty to inform was defective. Because the hospital did not fully disclose this risk, C lost the opportunity to decide whether or not to undergo the surgery, which constituted a tortious injury to the patient's rights (17).

There are also exceptions to the physician's duty to inform. For instance, suppose a patient suffers a major accident and is in a critically ill, life-threatening state, has lost consciousness, cannot communicate or express their wishes, and no close relatives can be reached. In such a situation, immediate emergency measures are required. Otherwise, the patient could die or suffer grave harm. In this scenario, under the exception clause for the duty to inform in Article 1220 of the Civil Code, medical measures may be taken immediately after approval by the person in charge of the medical institution or an authorized representative. This clause requires three conditions to be met: (a) an emergency situation; (b) the inability to obtain consent from the patient or close relatives; and (c) approval by the responsible official. A question remains: if close relatives can be contacted but refuse to give an opinion, or if their opinions conflict, may medical measures be administered to the patient? Out of respect for the principle of autonomy and to avoid inserting the medical institution into family decision-making, the legislature applies an “objective standard” in such cases. That is, if the patient's close relatives refuse to respond or cannot reach a consensus, the medical institution is not permitted to provide medical treatment to the patient (18).

For example, in a 2007 case involving the death of a patient L, the patient—who was pregnant—died along with her fetus because her husband X refused to sign the surgical consent form (19). In the aftermath of this case, the medical and legal communities strongly urged improvements to the laws governing emergency medical interventions. The State Council amended the surgical-consent provisions of the Regulations on the Administration of Medical Institutions, emphasizing that “when other special circumstances arise, the attending physician shall propose a medical treatment plan and implement it after obtaining approval from the person in charge of the medical institution or an authorized responsible person”. In a second amendment, this was further specified to: “In emergency situations, such as when rescuing a critically ill patient, if it is impossible to obtain the opinion of the patient or their close relatives, upon approval by the person in charge of the medical institution or an authorized responsible person, corresponding medical measures may be implemented immediately”. This provision serves as an exception to the exceptions for the duty to inform, further addressing gaps in the previous emergency exception.

## 3 Challenges posed by artificial intelligence to physicians' duty to inform

Medical AI has significantly improved the accuracy of diagnoses, eased shortages of medical resources, and enhanced surgical precision. It has, to some extent, reduced patients' cognitive burdens and mitigated physicians' personal biases. On the other hand, it has also eroded the physician's traditionally dominant role in disclosure, rendering the patient's right to informed consent somewhat hollow (20). To date, numerous legal disputes involving medical AI have arisen worldwide. Below, we discuss four representative cases to illustrate these challenges.

Case 1: A Chinese Pharmacy Chain's Fabricated Prescriptions. In Shanghai, one pharmacy chain used an AI system via an internet hospital platform to automatically generate 350 fake human albumin prescriptions over just 4 days, enabling illegal drug sales (21). A similar incident occurred in South Korea, where a man with no medical background operated an AI-powered website that provided prescriptions to users based on their described symptoms. He forged documents using a doctor's name to do so (22). These cases highlight AI's “dual-use” dilemma: when AI (or robots) fails to serve its designer's intended beneficial purpose and is instead exploited by bad actors for illegal ends, who should bear the resulting legal and ethical responsibilities?

Case 2: Class Action Lawsuit against Sharp HealthCare (California) (23). Sharp HealthCare was hit with a broad privacy lawsuit alleging that it used an AI-driven “ambient clinical documentation” tool to record doctor–patient conversations and transmit them to a third-party vendor without adequate consent. The complaint seeks statutory penalties on behalf of a class potentially exceeding 100,000 patients, as well as punitive damages, injunctive relief, and comprehensive correction of the purportedly inaccurate medical records.

Case 3: First Recorded Death in the UK from Da Vinci Robot–Assisted Heart Surgery (24). In 2015, during a robotic heart valve repair, the surgical robot sutured the wrong area and punctured a major artery, causing the patient's death within a week post-operation. At a hearing, the surgeon acknowledged that he had not fully disclosed the surgical risks to the patient and that he was insufficiently trained. This case exemplifies a scenario of “hybrid human-machine negligence”, which severely eroded patient trust. When a physician lacks sufficient control over a technology, technical flaws (such as “noise” interference) can amplify human errors, and ultimate liability in this case clearly fell on human negligence.

Case 4: Death Following Robot-Assisted Rectal Cancer Surgery in China (25). In 2023, patient D underwent a robot-assisted radical rectectomy. Subsequently, D was readmitted with a mechanical bowel obstruction and, despite emergency treatment, died. The cause of death was improper management of postoperative complications. A forensic assessment concluded that the hospital had underestimated the severity of the patient's condition, failed to adequately monitor the patient, and used an imperfect treatment method. Relying on this assessment, the court found that the hospital's lapses in care constituted medical fault and held the hospital 50% liable for damages. This case highlights the currently prevalent simplification of liability determination in judicial practice and the blurred allocation of responsibility between “AI-assisted” care and traditional human medical care.

### 3.1 The impact of composite actors on the physician–patient trust relationship

In the traditional model, the physician and patient formed a simple two-party relationship (“doctor–patient”). The physician, drawing on professional expertise, would disclose the patient's condition, treatment options, potential risks, and so on, and the patient would then decide—based on their own circumstances—whether to accept the recommended plan or opt for an alternative. This dual-subject relationship fully embodied respect for patient autonomy while still ensuring effective treatment. Once AI enters the picture, however, the classic dyadic relationship transforms into a composite “doctor + AI – patient” structure. This structural change underlies all the challenges discussed: it fundamentally undermines the singular foundation of authority and trust upon which the traditional duty of disclosure is built. Hippocrates once said, “Patients are like small boats adrift on a stormy sea, and the doctor must face them rationally”, and “A doctor's duty cannot be fulfilled by one individual alone; without the cooperation of patients and others, nothing can be accomplished” (26). These words highlight the collaborative, interdependent relationship between doctors and patients. Both traditional medical paternalism and unbridled patient autonomy have their limitations: the former overemphasizes the physician's authoritarian control of knowledge while ignoring the patient's autonomy over their own body, and the latter overemphasizes the patient's independence to the point that physicians may become emotionally detached, leaving patients “abandoned” in a void of autonomy. Beyond these two models of disclosure, a trust-based model of mutual participation has been proposed, with the aim of balancing patient autonomy and professional medical expertise (27).

In Case 1, AI was misused for fraudulent and other illegal purposes, completely corrupting the doctor–patient relationship that the technology was meant to support. Such incidents directly undermine the foundation of trust in the healthcare system. If AI can be readily used to forge prescriptions, patients' trust in prescription legitimacy, in physicians' professional judgment, and even in the entire medical regulatory framework will be severely damaged. In Case 3 and Case 4, the introduction of AI disrupted the legal relationship between physician and patient. Previously, the doctor and patient shared a direct, egalitarian, and complementary relationship: the physician would use their professional expertise to proactively disclose treatment plans and associated risks, and the patient would make a choice and final decision based on their individual circumstances. With AI's entry into healthcare, the foremost question becomes the agency of AI. If AI is regarded as an actor with decision-making agency, the patient must ask—can they trust a cold, impersonal machine? AI does have advantages in big data, often surpassing individual doctors in medical knowledge and case analysis. However, it remains uncertain whether AI can truly comprehend each patient's unique needs. Physicians must holistically evaluate a patient's personal characteristics, understanding, and other factors such as ethical beliefs, moral values, religious faith, cultural background, and philosophical outlook. Details that might seem insignificant to outsiders could be vitally important to the patient. Thus, doctors must perform meticulous, in-depth assessments for every patient (28). In traditional Chinese medicine, a core diagnostic approach known as “wang-wen-wen-qie” (observe, smell, ask, palpate) relies on the physician's active, in-person engagement: by closely observing a patient's physical and emotional state, asking targeted questions, and using professional knowledge, a physician guides the patient to fully articulate their condition. At present, it is unknown whether AI possesses a mature capability for such personalized observation, or whether it can meet the explanatory standards required for individual patients. If AI is not considered an independent decision-maker, the physician faces a different dilemma: whether or not to adopt the AI's recommendations. AI's data sources are vast, but also potentially biased. No doctor can master all medical knowledge to perfectly judge an AI's diagnoses or treatment suggestions. If physicians follow AI blindly, the physician's duty to inform risks being reduced to an “echo of the algorithm”, and patients' trust in their doctors could shift into an abstract trust (or mistrust) of AI technology. Traditional doctor–patient trust is built person-to-person, grounded in the physician's expertise, professional ethics, and empathetic connection. By introducing AI, we inject a need to gauge trust in technology. Particularly in the early phases of AI-assisted care, both doctors and patients tend to be wary of AI, which inevitably strains the trust dynamic in the doctor–patient relationship.

### 3.2 Medical AI expands the scope of physicians' duty to inform

Considering the scope of the duty to inform, we need to clarify who the obligated party is. The physician, as the party performing the medical intervention, is naturally the primary bearer of the duty to inform. Although regulations such as the Implementing Rules of the Medical Institutions Regulation and the Medical Accident Handling Regulation use the term “medical institution”, in practice the duty to inform rests with the physician who actually delivers the medical care (or the actual healthcare provider involved). Once AI-assisted diagnosis and treatment are introduced, the physician must explain not only their own assessment and treatment plan, but also assume the added responsibility of explaining the AI's recommendations. In a traditional doctor–patient relationship, the physician's explanation is limited to, “Why am I recommending Plan A?” In the composite relationship involving AI, the physician's scope of disclosure is expanded, adding an extra explanatory burden. The physician must, in addition to explaining their own plan, interpret the AI's suggestion—explaining to the patient the data or factors on which the AI's recommendation is based. Moreover, the physician must integrate the AI input into the final decision-making and explain to the patient why the AI's recommendation was either adopted or rejected. This unquestionably enlarges the scope of the physician's duty to inform, increases the explanatory burden on the physician, and raises the concern that the physician may be relegated to a mere instrument or “mouthpiece” of the AI.

Regarding the breadth of disclosure, Case 2 demonstrates that AI's involvement has introduced novel, forward-looking disclosure obligations. Healthcare providers must not only inform patients about their treatment plans but also, before using AI tools to handle data from doctor–patient interactions (such as recordings or analyses), explicitly inform patients about the scope and purpose of such use, where the data will go, and any third-party data sharing, obtaining separate consent for these steps. This goes far beyond the traditional scope of disclosure. In terms of depth of disclosure, if the physician cannot fully understand the AI's decision-making logic, interpreting the AI's recommendations becomes extremely difficult, if not impossible. Physicians may end up merely conveying the AI's treatment plan without being able to reasonably explain it, which profoundly weakens the effectiveness of informed consent. AI systems are often “black boxes” with limited transparency. If something goes seriously wrong, it's hard to trace the cause: a physician cannot verify the soundness of an AI's diagnosis, a patient cannot know the basis of the AI's advice, and regulators cannot easily evaluate the system's safety or fairness (29). Moreover, because AI's data collection can be imperfect, the AI's recommended treatment might be the product of biased data, resulting in algorithmic discrimination. One example is a “high-risk patient management” algorithm in a healthcare system that, for a long period, failed to include minority patients in certain beneficial programs. Even more concerning, some algorithms are deliberately adjusted using racial factors, leading to unfair outcomes for minority patients. For instance, a 2020 study in The New England Journal of Medicine found that such algorithms frequently underestimated the risk of conditions like kidney stones, heart failure mortality, and other health issues among African Americans (30). It is nearly impossible for physicians to assess the reasoning behind an AI's clinical judgment, which directly hinders their ability to explain AI-driven results to patients. To mitigate liability, physicians might engage in defensive disclosure—providing exhaustive technical details about the AI system and all potential risks. However, such information often exceeds the patient's comprehension and may render the patient's consent invalid due to misunderstanding. How to balance technological complexity with the attainment of truly informed consent has thus become a pressing new challenge for the duty-to-inform regime.

### 3.3 The current tiered classification regime for medical AI does not match physicians' duty to inform

Currently, according to various regulatory documents—including the Regulations on Supervision and Administration of Medical Devices, the Medical Device Classification Rules, the Medical Device Classification Catalog, the Procedures for Dynamic Adjustment of the Medical Device Classification Catalog, the Guiding Principles for Classification of AI Medical Software Products, and the Guiding Principles for Registration Review of AI Medical Devices—China employs three primary methods to classify medical AI: classification by risk level, classification by maturity level, and classification by degree of involvement.

First, medical devices are classified into three tiers based on risk: Class I devices are low-risk and can be safely and effectively managed through routine regulation. Class II devices carry moderate risk and require stricter regulatory control to ensure safety and efficacy. Class III devices are high-risk and demand special measures with very strict oversight to guarantee safety and effectiveness. In assessing a device's risk level, regulators consider factors such as the device's intended purpose, design characteristics, mode of use, conditions of use, and whether it comes into contact with the human body. Second, the regulatory category for medical software products is determined by considering the product's intended use and the maturity of its algorithm. For AI-driven medical software of low maturity in clinical use (i.e., not yet marketed or with safety and efficacy not fully validated), if it is intended to assist in clinical decision-making, it is regulated as a Class III medical device; if it is used for purposes other than decision support, it is treated as a Class II device. For AI medical software with high maturity (i.e., safety and efficacy well established), its regulatory category is determined according to the existing Medical Device Classification Catalog and related guidance documents. Third, from a functional perspective, AI medical devices are categorized as either decision-support or non-decision-support. Decision-support AI provides recommendations during clinical care to help users (such as healthcare professionals or patients) make medical decisions, effectively acting as the user's “assistant”. By contrast, non-decision-support AI provides only medical reference information without making decisions, effectively functioning as a “tool” for the user.

In Case 4, the “robot” used in the robot-assisted rectal cancer surgery would likely be classified as a high-risk Class III medical device. However, this device-focused risk classification does not directly address key clinical questions: In such a complex surgery, exactly how far does the physician's duty to inform extend? Must the physician explicitly explain the AI's specific role and limitations in the procedure, as well as any unique risks it introduces compared to a traditional surgery? The current classification regime offers no fine-grained guidance on these points. In general, China's regulation of medical AI remains rather rudimentary, and the existing AI classification system is misaligned with the requirements of the physician's duty to inform. This misalignment is partly because China's medical AI sector is a late starter that has evolved very rapidly, leaving legal norms lagging behind. It is also due to the inherent tension between fostering technological innovation and enforcing legal regulation—regulators must ensure that technology develops within the bounds of law, yet exercise caution so as not to unduly hinder technological progress.

### 3.4 Ambiguity in allocating responsibility

Research indicates that 13.72% of medical disputes are related to breaches of the duty to inform, making this the second most common cause of such disputes (31). This shows that a physician's failure to properly fulfill the duty to inform has become a major cause of medical litigation and has garnered widespread academic attention. When confronted with lawsuits stemming from inadequate disclosure, the key question becomes how to allocate responsibility.

Each of the four cases above illustrates the challenge of assigning liability. Case 1, for example, typifies the dual-use dilemma: responsibility potentially lies with the AI tool provider, the person who misused the tool, and the institution that failed to regulate its use. Current laws cannot clearly or fairly apportion liability among these parties, often making it difficult to hold anyone accountable or resulting in a liability gap. Case 2 involves a healthcare provider (as the deployer of AI), an AI developer (tool provider), and a third-party vendor. If a privacy violation occurs, is the liability due to the hospital's failure to adequately inform and supervise, or due to a design flaw in the developer's product that caused unauthorized data transmission? Such ambiguity complicates patient redress and judicial findings. Fundamentally, this uncertainty in liability is driven by the composite nature of the actors. Introducing AI into clinical care adds two new stakeholders: the AI's developer and its vendor. We now potentially have four parties at fault: the doctor who used the AI, the AI itself, the medical institution that implemented and oversaw the AI, and the AI's developer/vendor. Each of these four has plausible reasons and opportunities to be held liable, which makes the liability chain exceptionally complex. With so many actors possibly involved, determining each party's share of responsibility becomes extremely challenging. It is nearly impossible to precisely quantify each party's liability. For instance, consider a misdiagnosis resulting from multiple factors: bias in the AI's algorithm, the physician's failure to catch an obvious red flag, and the hospital's insufficient training of the physician. Deciding liability in such a multi-factor scenario poses an unprecedented challenge for the legal system.

Additionally, AI's involvement creates ambiguity in identifying physician negligence. Case 3 is a textbook example of blurred responsibility: the error was multi-factorial, stemming from both technological shortcomings (e.g., the robot's precision error or “noise” interference) and human failings (insufficient training, operational mistakes, inadequate risk disclosure by the doctor). Although ultimate liability in that case fell to the surgeon (since he admitted fault), courts face a formidable challenge in parsing and apportioning “technical defects” vs. “human error”. Placing all blame on the physician might mask the product liability that technology providers should bear. In Case 4, the court adopted a blunt approach by ruling that “the hospital bears half of the compensation liability”. The judgment did not delve into how the “robot assistance” contributed to the complication—was the critical failure in the surgical decision (human), the robot's execution (machine), or postoperative monitoring (human)? This factual and liability gray zone between “AI-assisted” and “human” care was glossed over, with no nuanced rule established to allocate liability in proportion to AI's involvement.

## 4 Comparative law review of medical AI applications

According to estimates, between 2018 and 2025 international bodies such as UNESCO, the European Commission, the EU High-Level Expert Group on AI, the European Group on Ethics in Science and New Technologies, the World Health Organization, and the OECD have collectively issued over twenty AI-related instruments (including conventions, guidelines, statements, reports, and declarations) (32). Major corporations and industry consortia—for example, Accenture, the Chinese Artificial Intelligence Industry Development Alliance, the Association for Computing Machinery, GE Healthcare, and Google—have likewise established their own data ethics guidelines. These international recommendations and industry standards display a great deal of similarity. In the following, we conduct a comparative law analysis focusing on influential international documents issued by key global institutions.

### 4.1 UNESCO's recommendations on the ethics of artificial intelligence

In 2017, UNESCO released the Report of Comest on Robotics Ethics (33). This document provides a comprehensive review of how robots are defined, their applications in society, and the ethical challenges they pose. In the sections on the design of medical robots and medical AI, the report—drawing on practical experiences with surgical robots, assistive therapy robots, rehabilitation robots, and care robots—affirms robots' capabilities in improving surgical precision, supporting rehabilitation, and reducing the burden of caregiving. At the same time, it expresses concerns regarding emotional and ethical risks, issues of responsibility and transparency, social fairness, and cultural differences. The report proposes that the manufacture and application of robots should embody seven ethical principles and values: human dignity, protection of autonomy, protection of privacy, the principle of non-harm, the principle of responsibility, the principle of beneficence, and the principle of justice. With respect to liability, the report highlights the problem of distributed responsibility, namely, the difficulty of making a clear allocation of responsibility among robot designers, engineers, programmers, manufacturers, investors, sellers, and users—especially in relation to the “dual-use” problem, where robots are not used to benefit humans as intended by their designers but are instead exploited by malicious actors for illegal or criminal purposes, raising the question of who should bear the corresponding legal and ethical responsibilities. In response, the report proposes two pathways: first, to develop technologies as far as possible to predict the impacts of robotic development; and second, to strengthen governance over the introduction of robots into society by treating it as a “social experiment” that must be carried out with caution.

In 2021, UNESCO issued the Recommendation on the Ethics of Artificial Intelligence (34). This Recommendation recognizes that artificial intelligence has a profound and dynamic impact—both positive and negative—on society, the environment, ecosystems, and all aspects of human life, including human thought. The Recommendation holds that while AI technology can bring significant benefits, realizing these benefits will also exacerbate issues that need to be addressed: conflicts and tensions arising from innovation, asymmetries in access to knowledge and technology, barriers to information access, gaps in human and institutional capacities, obstacles to accessing technological innovations, and the lack of adequate physical and digital infrastructure and regulatory frameworks. In light of this, the Recommendation puts forward four fundamental ethical values: “Respect, protect and promote human rights, fundamental freedoms and human dignity” “Environment and ecosystem flourishing” “Ensuring diversity and inclusiveness” and “Living in peaceful, just, and interconnected societies”. These are intended to uphold human autonomy, balance development with environmental sustainability, strengthen human solidarity, and promote stable social development. Building on these four values, the Recommendation proposes ten ethical principles, including the principle of proportionality and do no harm, the principle of safety and security, the principle of fairness and non-discrimination, the principle of sustainable development, the principle of privacy and data protection, the principle of human oversight and decision-making, the principle of transparency and explainability, the principle of responsibility and accountability, the principle of awareness and literacy, and the principle of multi-stakeholder governance and collaboration. Notably, the principles of human oversight and decision-making, transparency and explainability, and responsibility and accountability are highly relevant to medical AI. These three principles implicitly require that medical AI uphold human autonomy and be applied prudently within the limits of what is understood about the AI. In particular, the principle of “do no harm” explicitly demands that AI methods be suited to specific contexts and grounded in rigorous scientific basis. If a given decision would have irreversible or nearly irreversible effects, or involves matters of life and death, the final decision must be made by a human being.

### 4.2 Relevant EU provisions on artificial intelligence

In 2019, the EU's High-Level Expert Group on Artificial Intelligence released the Ethics Guidelines for Trustworthy AI (35). This guideline proposes that “Trustworthy AI” should focus on three components: it must be lawful, ethical, and robust, and these three aspects must be ensured throughout the AI system's entire lifecycle. While AI systems bring great benefits to individuals and society, they also introduce certain risks that should be mitigated with measures commensurate to the level of risk. The development, deployment, and use of AI systems should adhere to four ethical principles: respect for human autonomy, prevention of harm, fairness, and explicability, while recognizing and managing the potential tensions among these principles. Building on these four ethical principles, the guideline further sets out seven key requirements to ensure that the development, deployment, and use of AI systems comply with the standards of trustworthy AI: (1) human agency and oversight, (2) technical robustness and safety, (3) privacy and data governance, (4) transparency, (5) diversity, non-discrimination and fairness, (6) environmental and societal wellbeing, and (7) accountability. The guideline categorizes methods for achieving trustworthy AI during the design, development, and use phases into technical and non-technical methods, identifying ethical and legal norm integration in design as one type of technical method. As one approach to realizing trustworthy AI, the embedded design of ethics and law requires establishing a clear and precise link between the abstract principles that an AI system must follow and concrete implementation decisions. The core of this approach is to integrate compliance with norms into the very design of AI systems. Moreover, the guideline provides an AI assessment checklist based on the aforementioned seven key requirements, breaking down each requirement into numerous assessment items, which makes it highly operational in practice.

In 2024, the EU introduced the world's first comprehensive regulatory framework for artificial intelligence, the Artificial Intelligence Act (36). This Act adopts a “human-centric” AI development strategy and categorizes AI system risks into four levels: unacceptable risk, high risk, limited risk, and minimal risk, imposing different compliance obligations for AI systems at each risk level. Unacceptable risk scenarios include applications such as manipulation of human behavior, exploitation of specific vulnerabilities, social scoring, biometric categorization, and real-time remote biometric identification. According to Article 6 of the Act, an AI system is deemed high-risk if it meets both of the following conditions: (1) the AI system is intended to be used as a safety component of a product, or the AI system itself is a product listed in Annex I of the Act that is regulated by EU harmonization legislation; and (2) if a product's safety component is an AI system, or the AI system itself is a product, it must undergo third-party conformity assessment under the EU harmonized legislation listed in Annex I, to ensure compliance before the product is placed on the market or put into service. In addition, AI systems listed in Annex III of the Act are also regarded as high-risk, including specific AI systems in areas such as critical infrastructure, education and vocational training, employment, access to and enjoyment of essential private services and public services and benefits (e.g., medical services), as well as in law enforcement, border control, administration of justice, and democratic processes. For limited-risk AI systems, the Act imposes certain transparency obligations: providers and deployers of such AI systems must ensure that users are aware they are interacting with an AI system. For the vast majority of other AI systems that pose minimal risk or virtually no risk, the Act does not intervene. The Act places regulatory focus on high-risk AI, setting out detailed compliance requirements in areas such as risk management, data governance, technical documentation, record-keeping, transparency, human oversight, accuracy, robustness, and cybersecurity for high-risk AI systems. Furthermore, the Act provides a detailed list of compliance obligations for various participants across the high-risk AI system supply chain, including providers, importers, distributors, and deployers.

### 4.3 WHO guidance on medical artificial intelligence

In 2021, the World Health Organization (WHO) released for the first time Ethics and governance of artificial intelligence for health: WHO guidance (37). This guidance stresses that humans should always retain final control over medical decisions. In areas such as diagnosis, prognosis, and treatment recommendations, medical AI should serve as an assistive tool to enhance clinical judgment, rather than replacing the autonomy of doctors or patients. The WHO expert group identified six core principles: (1) Protect autonomy; (2) Promote human wellbeing, human safety, and the public interest; (3) Ensure transparency, explainability, and intelligibility; (4) Foster responsibility and accountability; (5) Ensure inclusiveness and equity; (6) Promote AI that is responsive and sustainable. These principles must be observed not only by healthcare personnel when using AI systems to perform clinical tasks or take over clinical work traditionally done by humans, but also by the programmers who design and develop such AI technologies, who bear corresponding ethical responsibilities.

The WHO guidance places particular emphasis on safeguarding human autonomy. The principle of autonomy mandates that any extension of machine autonomy must not undermine human autonomy. In the context of healthcare, this means that humans should always remain in full control of medical systems and decisions. AI systems should be designed to assist humans (whether healthcare providers or patients) in making informed decisions. In practice, this may involve deciding whether to use an AI system for a given clinical decision, calibrating the level of human independent judgment and decision-making, and developing AI technologies capable of providing tiered evaluations of decisions.

Regarding accountability, the field of AI faces what is known as the “control problem”. Accountability for medical AI is a multi-level, multi-actor systemic issue, requiring a clear and fair liability framework among technology developers, healthcare institutions, regulators, and clinicians, rather than unfairly placing the entire burden on frontline doctors. If only physicians are held liable, technology developers and companies may evade responsibility, and doctors could become “scapegoats” held accountable for technical decisions beyond their control. When AI is integrated into the overall healthcare system, allocating responsibility becomes exceedingly complex. The guidance favors an approach of dividing responsibility based on actual circumstances: developers, healthcare institutions, and government regulators may all be implicated, forming a shared liability arrangement instead of any single party bearing full responsibility.

In 2024, the WHO released Ethics and governance of artificial intelligence for health: Guidance on large multi-modal models (38). Large multi-modal models (LMMs), as a form of generative AI, can process various inputs such as text, images, and biological data and produce a range of outputs. They are being widely applied in healthcare, research, public health, and other fields. The use of LMMs in diagnosis and clinical care carries five major risks: inaccurate information, data bias, automation bias, skill degradation, and informed consent risks. With the deployment of LMMs, patients can more conveniently explore their own health issues using these models, but they also face enormous risks brought by the technology. In addition to problems like misinformation, emotional manipulation, and data discrimination, the use of LMMs by patients or caregivers could fundamentally alter the doctor–patient relationship, reducing interaction among clinicians, non-professional personnel, and patients. Patients or caregivers might even decide to rely entirely on LMMs for prognostication and treatment, thereby diminishing or eliminating appropriate reliance on professional medical judgment and support.

This WHO guidance analyzes risks and provides recommendations for three stages of LMM deployment—design & development, provision, and deployment:

(a) Design & Development Stage: Developers of general foundation models should address eight major risk areas (via government laws and regulations, among other avenues): bias, privacy, labor rights issues, carbon and water footprint, misinformation/hate speech/disinformation, safety and cybersecurity, preservation of human cognitive authority, and exclusive control of LMMs. To mitigate these risks, developers should proactively assemble professional teams during the design and development phase, strengthen data quality management, embed ethical values into design, and adopt environmentally friendly design practices. Governments should guide responsible technology development through legislation, regulation, and public investment—specifically by strengthening data protection laws, establishing technical standards and certification mechanisms, promoting public infrastructure development, and instituting environmental impact and transparency requirements.
(b) Provision Stage: The main risks at this stage include unclear liability attribution, systemic and application-specific risks, and regulatory gaps. Governments should control these risks by establishing oversight and evaluation frameworks, enhancing transparency and disclosure, strictly enforcing data protection laws, instituting human-rights-based assessment mechanisms, clarifying the applicability of medical device regulations, and leveraging consumer protection laws. Developers and providers should in turn proactively limit the use of LMMs and assume shared responsibility to prevent risks.
(c) Deployment Stage: Key risks at this stage include unpredictable usage outcomes, propagation and amplification of risks, and issues of accessibility and equity. Developers and providers need to carry out ongoing post-deployment auditing and monitoring, as well as responsibilities for timely correction and disclosure of issues. Deployers (e.g., healthcare institutions) must exercise caution when applying LMMs, clearly inform patients of the risks and limitations, and ensure the use of LMMs is fair and accessible. Governments should provide systemic support and continuous training by establishing oversight associations, guiding public-sector behavior, and raising public awareness. The accountability stage is the final critical link to ensure ethical principles are implemented and to protect victims. Given the multi-party involvement and the risk of blame-shifting, an effective accountability mechanism is recommended, centered on compensating victims and creating a lasting effective system of deterrence and incentives.

### 4.4 Commentary

The above international documents all highlight the dual nature of AI as both an opportunity and a risk, and they share a philosophy of maintaining balance in AI development and oversight.

- a. Fundamental commitment to human autonomy. At the current stage of AI development, virtually all these documents explicitly endorse a “human-centric” value framework, emphasizing that humans should retain final decision-making power. In other words, AI development and utilization should uphold the primacy of human dignity and autonomy, maintain transparency and explainability of AI systems, and ensure that ultimate allocation of responsibility is grounded in scientific and practical considerations. Especially in the domain of medical AI—which involves the most fundamental of human interests, life and health—these documents make particularly cautious provisions. This reflects the global community's firm stance on defending human agency in the face of technological uncertainty.
- b. A risk-based, tiered regulatory approach. The international reports and regulations above have generally followed a “risk-centric” AI classification system. Confronted with AI's rapid rise and the great uncertainties it brings, international bodies have reached an implicit consensus on balancing risk prevention with encouraging innovation. In terms of risk management, there is a worldwide trend from broad ethical consensus toward more fine-grained regulatory practice. In particular, the EU's Artificial Intelligence Act explicitly defines risk levels for AI and sets detailed requirements for high-risk areas. This “calibrate regulatory stringency to risk level” approach avoids the stifling of innovation that can come from a blunt one-size-fits-all policy, while precisely focusing regulatory resources on areas most likely to significantly impact fundamental rights. It provides a highly practical legislative model for the world.
- c. A multi-party shared liability framework. AI—especially medical AI—involves a complex supply chain with many actors (designers, developers, providers, deploying institutions, clinicians, patients, etc.), which challenges traditional tort law approaches to assigning liability. All three major international organizations acknowledge this complexity and have independently moved away from the simplistic notion of a single entity bearing all responsibility. Instead, they advocate establishing a clear and fair framework of shared responsibility among multiple parties. They suggest dividing responsibilities according to the various stages of AI's lifecycle from development to deployment, and apportioning liability to each participating party based on the extent of their substantive involvement. The liability mechanism must align with the AI system's lifecycle. For example, there should be clear delineation and linkage of a developer's “design responsibility”, a provider's “compliance responsibility”, a deploying institution's “duty of careful selection and operation”, and a clinician's “professional final judgment responsibility”. The future liability framework will likely be a multi-tiered hybrid model based on fault and role-specific duties, potentially supplemented by mechanisms like insurance or compensation funds to distribute risk effectively.

## 5 Recommendations

Because medical AI is built upon artificial intelligence technology, it possesses all the attributes and risk characteristics of AI. In the face of the challenges that medical AI poses to physicians' duties to inform, we must first clarify whether medical AI possesses legal subject status. Only then can we undertake a typological analysis based on its characteristics, and, in conjunction with concrete use scenarios, delineate the specific scope of physicians' duties to inform after the involvement of medical AI.

### 5.1 Medical AI does not yet possess legal personhood

Clarifying how a physician's duties to inform change after the introduction of medical AI inevitably requires addressing a fundamental question: does medical AI possess personhood attributes? From a comparative law perspective, countries hold different views on the legal status of medical AI, which can be generally divided into three positions: the affirmative view, the negative view, and the intermediate view. In the legal academy as well, scholars are essentially split into these three distinct stances.

As shown in Table 1, the affirmative view advocates granting AI a fictitious independent legal personality, analogous to how legal subjects have expanded from natural persons to corporations and other organizations—effectively allowing AI to participate in legal relations through a legal fiction. The negative view, by contrast, does not recognize AI as having legal personhood or subject status. The intermediate view takes a cautious or conditional stance on whether to endow AI with subject status, and it includes variants such as the limited legal personhood theory and the electronic personhood theory.

**Table 1:** Comparison of main doctrines on the legal personhood of artificial intelligence.

**Dimension**	**Affirmative view**	**Negative view**	**Intermediate view**
Core claim	AI should be granted an independent legal personality through legal fiction and become a legal subject.	AI should not be treated as a legal subject; it should be defined as a tool.	A cautious or limited-recognition approach to AI personhood.
Main reasons	a. Value extension: recognizing rational AI can be framed as respecting human dignity (48) b. Historical analogy: the scope of legal subjects can expand (e.g., legal persons) (49) c. Technological development: strong AI/AGI may develop autonomy and rationality (50)	a. Lack of biological basis: no human body/brain/emotions (51) b. Lack of subjective basis: no moral judgment; cannot comprehend consequences (52) c. Practical needs: defining AI as a tool clarifies liability and protects patients and medical order (53)	a. Limited personality: some autonomy, but limited capacity for responsibility (54) b. “Electronic person”: a special legal subject with limited rights and obligations in a defined scope (55)
Representative claims	Establish AI subject qualification by analogy to corporate personhood via legal fiction.	Treat AI as a product/tool; assign responsibility to developers, users, or institutions.	Grant quasi-civil-subject status through procedures, often to protect AI-generated outputs rather than to recognize AI as a full subject.
Potential stance toward medical AI	Strong AI in medicine could become an independent liable subject.	Medical AI should be treated as advanced medical device/tool; responsibility should be traced to humans.	In highly autonomous medical scenarios, AI might bear limited responsibility, but human oversight remains indispensable.

In view of the current stage of development of medical AI, it is premature to grant it legal subject status; it should instead be treated as an advanced medical device. Historically, legal personhood has indeed continually expanded—evolving from natural persons to corporate entities and even beyond the biological notion of a “person” (39)—but at this stage, although AI's deep learning capabilities are increasingly approaching human thinking patterns, it has not yet reached the technological “singularity” (40). Especially in the medical domain, which concerns life and health, we must approach AI with great caution. In truth, AI lacks free will and rationality: an AI cannot spontaneously generate consciousness, nor can consciousness be forcibly implanted into it from outside, so it cannot possess autonomous will (41). Present-day AI is merely an extension of human rationality and does not surpass the integrality of human thought. It falls short of achieving true rational cognition (42). One scholar has noted that even if a so-called “strong AI” emerges in the future, it would not have genuine free will or rationality, but would only be “an enhanced version of a weak AI entity” (43). Moreover, current AI lacks emotions and desires—the human-like motivational drivers of behavior. Humans are desiring subjects; only beings with emotions and desires can bear responsibility in balancing interests (44). In the medical context, an AI's algorithms and decision-making processes rely heavily on data inputs and program settings provided by humans, and its “actions” are essentially the results of executing code rather than expressions of autonomous will. To regard AI at this point as an independent legal subject would not only be logically inconsistent, but would also lead to confusion in the attribution of legal responsibility and potentially harm patients' legitimate rights and interests.

### 5.2 Establishing a four-level classification system aligned with physicians' duty to inform

The risk-based AI classification schemes proposed by international organizations certainly have merits, but it should be noted that those frameworks are formulated from the perspective of the AI industry as a whole, as cautious recommendations to address future uncertainties and significant ethical and legal risks. In contrast, medical AI for assisted diagnosis and treatment has already achieved relatively mature results at present. Therefore, under the guidance of the existing risk-tier framework, it is necessary to propose a classification system centered on mode of application, tailored to medical AI's concrete usage scenarios, in order to tackle more specific and well-defined practical challenges. Moreover, the traditional classification methods based on risk level, algorithm maturity, and degree of human involvement have proven inadequate in addressing the challenges that medical AI poses to physicians' duties to inform (45). For these reasons, this paper constructs a classification framework based on two dimensions—clinical decision-making autonomy and degree of doctor–patient relationship impact—to directly respond to the ethical and legal dilemmas arising in medical AI applications regarding “what the physician should inform, to whom responsibility is owed, and how to obtain consent”.

**Figure 1:**
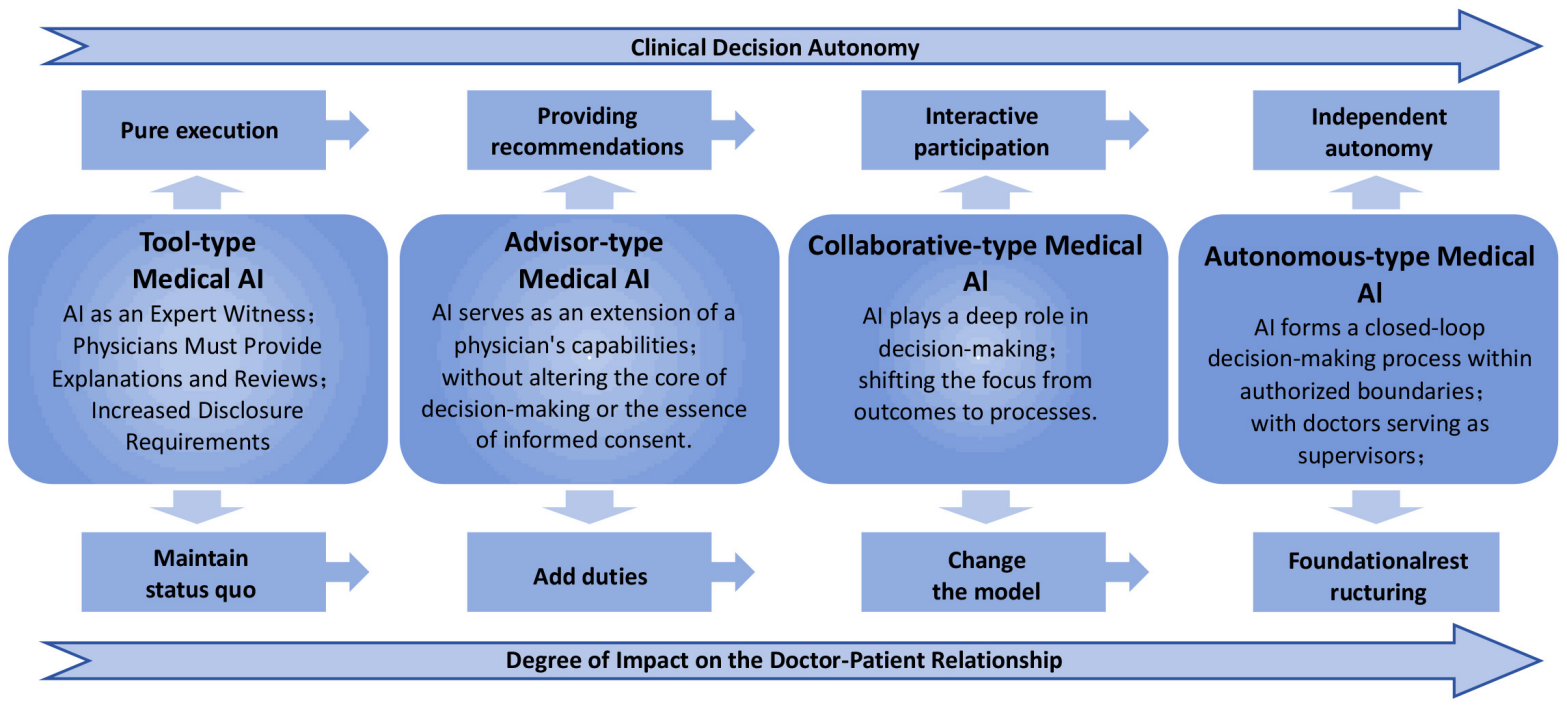
Classification framework for medical artificial intelligence: based on clinical decision autonomy and impact on doctor–patient relationships.

As shown in Figure 1, the upper axis (Clinical Decision Autonomy) illustrates a continuum in clinical decision-making by AI, ranging from “fully executing instructions” to “making and implementing decisions independently”. Tool-type medical AI strictly follows directives and carries out preset, standardized tasks, exhibiting no independent judgment in clinical decision-making—its “actions” are entirely predictable, programmatic outputs. Advisor-type medical AI, on the other hand, can independently analyze data and generate diagnostic or therapeutic suggestions, demonstrating an initial level of cognitive autonomy, though this autonomy is confined to the realm of “assistance” and “reference”. Collaborative medical AI becomes a dynamic interactive partner throughout the entire course of diagnosis and treatment, engaging in joint decision-making with the physician. Its autonomy is manifested in complex, context-dependent interactive participation. Autonomous medical AI is a system that, under certain conditions, can independently complete the full loop of “perception–analysis–decision–execution” without real-time human intervention, operating within a scope authorized in advance.

The lower axis (Degree of Doctor–patient Relationship Impact) depicts how AI's involvement disrupts the traditional doctor–patient relationship, communication patterns, and the complexity of the physician's duties to inform. Tool-type medical AI does not alter the conventional doctor–patient dyadic interaction and trust model. The doctor–patient relationship essentially remains unchanged. Advisor-type medical AI increases the physician's explanatory obligations: the doctor must provide additional explanations to the patient regarding the AI's involvement, the uncertainty of its conclusions, and the reasons for accepting or rejecting the AI's suggestions—informing thus becomes a new burden for the physician. Under a Collaborative medical AI, the focus of the doctor–patient relationship shifts from the physician unilaterally informing the patient of “results” to a mutual understanding of the “process”. The physician must communicate with the patient about how decisions are being made collaboratively between human and AI and about any potential uncertainties. During this interaction, trust and responsibility begin to be shared jointly by doctor and patient. Autonomous medical AI, in contrast, fundamentally restructures the doctor–patient relationship: its foundation shifts from interpersonal trust to a clearly defined legal contract. The crux of this arrangement is the patient's explicit prior authorization of the AI's autonomous actions, rendering the doctor–patient relationship highly formalized and rule-based.

As shown in Table 2, focusing on clinical decision autonomy and degree of doctor–patient relationship impact, and taking into account the AI's level of autonomy, the division of labor between medical AI and physicians, and the extent of AI's involvement, medical AI can be categorized into four types: tool-type, advisor-type, collaborative, and autonomous.

**Table 2:** Four-level classification of medical AI and its impact on physicians' duties to inform.

**Category**	**Physician's core role**	**Focus of the duty to inform**	**Illustrative cases**
Tool-type AI	Final decision-maker; physicians retain full control and use AI primarily as an efficiency tool.	Routine disclosure: explain that AI is used as an auxiliary component in the workflow; special authorization is generally unnecessary.	Voice-to-text electronic medical record systems; imaging pre-processing software.
Advisor-type AI	Discretionary decision-maker and reviewer; physicians decide whether to adopt AI suggestions and must professionally review outputs.	Explanation and record-keeping: disclose AI involvement and explain/record the reasons for adopting or rejecting AI suggestions.	AI imaging decision platform for acute stroke e.g., differential analysis and risk assessment for hemorrhage vs. ischemia.
Collaborative AI	Co-decision-maker and explainer; physicians and AI jointly shape decisions, with emphasis on process oversight and patient communication.	Process transparency: shift from “outcome” to “process”—explain how human–AI collaboration works and its uncertainties.	ICU prediction and intervention systems where AI generates alerts and suggestions and smart devices execute after physician confirmation.
Autonomous AI	Supervisor and intervener; the physician role shifts from real-time control to ex ante authorization and ex post supervision.	Specific authorization: clearly disclose autonomous decision functions; obtain patient's specific authorization and clarify supervision duties.	Highly automated surgical robots in defined scenarios; fully automated diagnostic modules based on deep learning.

a. From the perspective of increasing AI involvement, tool-type AI primarily performs standardized tasks (such as record-keeping or data preprocessing). Decision-making authority remains with the physician, and the existing doctor–patient decision-making process is unchanged. Tool-type AI has the smallest impact on the physician's duties to inform: the physician follows the normal procedure and only needs to inform the patient of the AI's auxiliary role in the workflow.
b. Advisor-type AI refers to a medical assistant system capable of providing auxiliary analysis and decision suggestions. For example, the acute stroke intelligent imaging decision platform at Beijing Tiantan Hospital can automatically differentiate intracerebral hemorrhage from ischemia based on CT scans, clearly delineate hemorrhage regions, and further indicate the type of hemorrhage and the probability of hematoma expansion (46). Advisor-type AI can assist physicians in disease understanding and case analysis, but it functions solely as an advisory aide, the physician maintains discretion over whether to accept its suggestions and bears a duty to review them. In practice, this means the physician must disclose the AI's involvement in diagnosis and treatment, explain the reasons for adopting or rejecting the AI's suggestions, and document these decisions. The physician still retains final decision-making power and responsibility.
c. Collaborative AI denotes a dynamic decision-support system that interacts with physicians. Collaborative AI can cover the entire care process, using large models to analyze conditions and provide diagnoses, treatment plans, and personalized medication recommendations. For example, Tianjin Haihe Hospital's intensive care prediction and intervention system automatically generates a dynamic risk assessment chart and corresponding intervention suggestions after the AI issues a red alert, Once the physician confirms the intervention plan, a smart infusion pump administers medication accordingly, and nurses receive alarms for follow-up monitoring (47). Collaborative AI has a greater impact on the physician's duties to inform, shifting the focus from the “outcome” to the “process”. Because AI has a “black box” nature and data limitations, the physician must explain to the patient how decisions are being made collaboratively between human and AI and what potential uncertainties exist, in order to safeguard the patient's right to know and autonomy. Ultimately, with adequate disclosure, part of the decision-making and corresponding liability is transferred to the patient.
d. Autonomous AI is the highest-intelligence, most independent form of medical AI, capable of autonomous and deep learning and of performing clinical tasks on its own in certain scenarios. In this model, the physician shifts from being an “intervenor during the process” to the roles of “pre-authorizer” and “*post hoc* supervisor”. Prior to treatment, the physician must ensure the AI system is suitable for the current patient and verify the patient's data; during treatment, if the system issues alarms or anomalies, the physician must promptly intervene and take over; after treatment, the physician needs to audit the AI's activity logs to confirm its actions stayed within expected and authorized bounds. Use of an autonomous AI requires the patient's specific prior authorization, and its risks and limitations must be explicitly disclosed to the patient. Any medical liability arising from the AI must be distinguished based on whether it exceeded the patient's authorized scope: if it went beyond the special authorization, the AI developer bears primary responsibility and the physician has secondary liability within the scope of their informing duty; if it did not exceed the authorized scope and the physician fulfilled the duty of prior disclosure, then the patient assumes primary responsibility and the developer bears liability commensurate with its degree of fault. Furthermore, the patient always retains the right to refuse the involvement of autonomous AI in treatment, and the physician should respect the patient's wish and adjust the treatment plan accordingly. The physician must also always have the ability to halt AI-driven treatment and take over the medical decision-making at any time.

### 5.3 Establishing a graded accountability system matched to the four-level AI framework

Building on the above four-tier classification framework, it is necessary to further clarify physicians' duties to inform and the principles of liability allocation under each type of AI assistance. The core principle is that the allocation of responsibility should be directly proportional to the degree of clinical decision control retained by the physician, and inversely proportional to the AI algorithm's “black-box” opacity. A physician cannot be absolved of ultimate responsibility for professional judgment simply by using an AI, but the specific form and emphasis of the physician's liability will change depending on the type of AI involved.

As shown in Table 3, the use of tool-type AI does not alter the fundamental structure of a physician's duties to inform. The physician is not required to specially explain to the patient the extent of involvement or the functions of the medical AI. Even if mentioned, it can be disclosed once as part of the routine workflow explanation, and ultimately the full responsibility for any failure to inform remains with the physician. This type of AI can be regarded as an extension of the doctor's senses—its main task is to re-present objective facts, and it is aimed primarily at assisting the physician rather than the patient. The physician bears complete responsibility for all information ultimately presented to the patient, with the key element being the duty to review. For example, if an AI transcribes a medical record incorrectly and the physician fails to catch and correct it when signing off on the record, leading to a medical error, the physician must bear full responsibility. In other words, the physician does not need to explicitly tell the patient “I used a voice transcription system”. Even if this is mentioned, it is only a procedural note and does not involve any transfer of legal risk.

**Table 3:** Framework of physician duties to inform and liability allocation under the four-level medical AI classification.

**Category**	**Principle of allocation**	**Attribution of responsibility**	**Example**
Tool-type AI	Physicians bear full responsibility.	Physicians are fully responsible for all information presented to the patient and for the final medical decision.	If an AI system mis-transcribes records and the physician fails to review, causing harm, the physician bears
Advisor-type AI	Dual-track allocation.	a. Physicians are responsible for clinical decisions. b. Developers are responsible for inherent algorithmic defects (within claimed performance).	In an imaging decision platform, the physician must explain why the AI hemorrhage-risk assessment is adopted.
Collaborative AI	Responsibility shifts toward patient decision-making (with adequate disclosure).	With sufficient disclosure, patients assume responsibility for the final choice based on understanding; developers/institutions ensure process compliance and explainability.	In an ICU prediction system, physicians must explain how human–AI collaboration shapes the intervention plan.
Autonomous AI	Allocation based on scope of authorization.	[a] If AI exceeds authorization: developers bear primary responsibility; physicians bear secondary supervisory responsibility. If AI stays within authorization: patients bear primary responsibility; developers bear fault-based proportional responsibility.	For autonomous surgical robots, physicians must ensure a specific authorization document for AI autonomous operation is signed.

The use of advisor-type AI introduces a partial shift in liability: the physician retains responsibility for the clinical decisions, while the AI developer assumes corresponding legal liability for any medical mishaps caused by technical issues with the AI. In this scenario, the AI is participating in diagnosis and treatment as an “expert witness”, providing recommendations of diagnostic significance. When fulfilling the duty to inform, the physician must tell the patient that the AI serves only an assistive role and that its conclusions have uncertainties; nevertheless, the final judgment remains in the physician's hands. For the physician, when an advisor-type AI is involved in care, they must carry out a professional review obligation, using their medical expertise to evaluate the data provided by the AI. Regarding the standard of disclosure, the physician should meet the professional standard of a rational physician. If a physician causes a medical accident by blindly following the AI's advice, the physician will bear legal responsibility. The AI developer's responsibility is to ensure that the algorithm's performance metrics, as claimed at the time of its approval or certification, are truthful and reliable. If due to inherent defects of the algorithm (within its claimed performance scope) a physician is unable to detect an error despite fulfilling their review duty, or if the AI has a significant design defect beyond its claimed scope, then the developer should bear legal liability.

After a collaborative AI is introduced into clinical practice, the physician must proactively and clearly disclose the AI's deep involvement and its inherent “black box” characteristics, and explain the collaborative decision-making model to the patient. The relationship between the physician and the AI becomes one of consultative collaboration. In this context, the physician may not fully understand the AI's reasoning and decision basis, but they still possess the professional ability to evaluate the AI's final recommendations. Unlike in the advisor-AI scenario, at this stage the physician's role shifts from “professional reviewer and final decision-maker” to “process supervisor and explanatory guide”. The physician's liability focus moves away from making the final decision toward supervising the process and communicating effectively—guiding the patient to understand the rationale behind the AI's output. In terms of legal liability, since collaborative AI has a higher degree of autonomy and advanced learning capability, it begins to exhibit certain traits of an independent agent, and the physician no longer bears vicarious liability for the AI. The legal responsibility linked to the physician's duty to inform is transferred to the patient through the shift in decision-making power: the physician no longer retains final decision authority, and as long as they have provided thorough explanation and guidance, their duty of disclosure can be considered fulfilled. The AI developer, meanwhile, must furnish information on the algorithm's underlying operational principles and feasibility, and ensure that the AI's decision-making process adheres to medical ethical standards.

The introduction of autonomous AI causes a radical shift in liability allocation: the developer assumes primary responsibility for the AI's actions, while the physician only bears responsibility for oversight and intervention. In this situation, the crux of the physician's duty to inform is to clearly tell the patient that key parts of the treatment will be carried out autonomously by AI, and to obtain the patient's explicit consent (special authorization) for this arrangement. At this highest level, the AI can form a closed-loop in the treatment process, effectively replacing portions of the physician's decision-making and execution functions. The physician's role undergoes a fundamental change, from “operator” to “supervisor”. In practice: (a) Before treatment, the physician must ensure the AI system is appropriate for the patient and verify the patient's data; (b) During treatment, if the system issues an alert or behaves abnormally, the physician must promptly intervene and manually take over; (c) After treatment, the physician needs to review the AI's execution logs to confirm that its behavior stayed within expected bounds and within what was disclosed and authorized by the patient beforehand. If a medical dispute arises from an autonomous AI, the first step is to determine whether the AI acted beyond the scope of the patient's special authorization. If it exceeded the authorized scope, the AI developer should bear primary responsibility for the AI's autonomous actions and their direct consequences, and the physician's liability is secondary, limited to the scope of their duties to inform. If the AI did not exceed the authorized scope and the physician had fulfilled the duty of prior disclosure, then the primary responsibility is voluntarily assumed by the patient, and the AI developer bears liability proportional to its degree of fault.

## 6 Conclusion

The normative purpose of physicians' duties to inform is not to punish physicians, but to remove barriers created by knowledge gaps, to maintain an equal footing for doctor–patient communication, and to ensure the patient's autonomy over their own body, thereby achieving the best therapeutic outcome within the scope of the patient's consent. With the advent of medical AI, the physician's duty to inform has been endowed with new and more complex connotations: physicians must not only disclose new risks and uncertainties and address the novel information asymmetries introduced by AI, but also redefine the boundaries of human doctors' responsibilities. Therefore, the restructuring of the duty to inform must always uphold these aims, establishing a standardized duty of care that is typified and phased. By classifying medical AI into tool-type, advisor-type, collaborative, and autonomous categories, we can construct a detailed and effective framework for duties to inform and liability allocation, one that is better adapted to evolving societal trends. More importantly, this four-level classification scheme helps strike a balance between technological innovation and legal oversight: it ensures that medical AI operates within legal and ethical boundaries at all times, without unduly over-penalizing developers in a way that would stifle the development of new technologies.

## Data Availability

The original contributions presented in the study are included in the article/supplementary material, further inquiries can be directed to the corresponding author.
